# An Insight into the Solid-State Miscibility of Triacylglycerol Crystals

**DOI:** 10.3390/molecules25194562

**Published:** 2020-10-06

**Authors:** Jorge Macridachis-González, Laura Bayés-García, Teresa Calvet

**Affiliations:** Departament de Mineralogia, Petrologia i Geología Aplicada, Facultat de Ciències de la Terra, Universitat de Barcelona, 08028 Barcelona, Spain; laurabayes@ub.edu (L.B.-G.); mtcalvet@ub.edu (T.C.)

**Keywords:** triacylglycerol, polymorphism, phase behavior

## Abstract

The crystallization properties of triacylglycerols (TAGs) strongly determine the functional properties of natural lipids. The polymorphic and mixing phase behavior of TAG molecules have long been, and still are, a hot topic of research with special relevance for the cosmetic, pharmaceutical, and food industry. To avoid the difficulties arising from the study of whole real fats, studies at the molecular level on mixtures of a limited number of TAGs has become an indispensable tool to identify the underlying causes of the physical properties in lipid systems. In particular, phase diagrams of binary mixtures of TAGs exhibiting a different degree of heterogeneity (monoacid or mixed fatty acids; molecular symmetry; the presence of *cis* or *trans* double bonds) have resulted in a significant breakthrough in our knowledge about structure–interaction–function relationships. The present work aims to provide an overview of the main reports regarding binary and ternary TAG systems, from the early studies to the most recent developments.

## 1. Introduction

The sensory properties (texture, rheology, appearance, melting behavior) displayed by lipid-based food products are largely determined by fat crystallization processes [1,2,3,4], in which triacylglycerol (TAG) molecules play a crucial role as major components. The overall macroscopic mechanical and rheological properties are influenced by the microstructure of the tridimensional network formed by lipid aggregation, whose strength relies on the volume of crystallized mass, crystal morphology and intermolecular interactions [5,6]. In turn, complex properties of the network, such as crystal aggregation, crystalline size and shape, solidification behavior, and thermal stability, are related to the physical properties of TAGs through their polymorphism and mixing phase behavior [7,8,9]. Thus, a deep understanding in lipid crystallization processes is required for optimal control for many food industrial applications: manufacture of end products, including spreads, shortenings, and confectionery; separation of fat fractions with specific physical and melting properties during oil fractionation; and, the development of lipidic materials with tailored functionalities through fat blending [5,10,11,12,13,14,15,16].

Polymorphic occurrence and mixing phase behavior of TAGs are largely dictated by molecular interactions in order to stabilize aliphatic chains, glycerol groups, and methyl-end stacking, which, in turn, rely on the nature of the esterified fatty acids (degree of unsaturation, presence of *cis* or *trans* double bonds, length of the hydrocarbon chains) and their distribution within the molecule [17,18]. In lipids, polymorphism is of monotropic nature and metastable forms tend to transform into more stable ones mainly via melt-mediation or through the solid-state. According to the type of subcell structure of TAGs, which is defined by the cross sectional packing of aliphatic chains, three basic polymorphs, namely α, β’, and β, are typically found [19]. In addition, different chain-length structures, understood as the number of acyl chains or leaflets in the crystal lattice *c*-axis parameter resulting from the head-to-tail stacking of TAGs during the formation of unit lamellas, may arise depending on the physicochemical properties of component fatty acids. Identical properties between the three fatty acid moieties lead to double chain-length structures (2L) [20,21,22], whereas dissimilarities might increase steric hindrance, preventing their concurrent packing in the same lamellar plane and triple chain-length (3L) structures arise as a result of chain sorting [23,24,25]. Nevertheless, more complex polymorphism with additional forms and larger chain-length structures may occur in mixed-acid TAGs [26,27,28,29].

In addition to the intrinsic properties of TAG molecules, the crystallization and polymorphic behavior of lipids have shown to be strongly dependent on processing conditions [14], such as the application of shear rates [30,31,32,33], sonication [34,35,36] or emulsification [37,38,39,40]. Moreover, special attention has been paid to the influence of dynamic thermal treatments of cooling and heating on the polymorphic crystallization and transformation of TAGs, their mixtures, and more complex fats [41,42,43,44,45,46,47,48,49], which become of special relevance from an industrial point of view [28,50].

Because complex fats consist of multi-component mixtures of TAGs, their physical behavior must be understood as the synergistic effect between TAG components over polymorphism and the different mixing states within. However, the number of different molecules involved is usually too high, even in the order of several hundred [51,52]; and, therefore, the study of complex fats to establish relationships between physical behavior and molecular interactions results in being rather complicated. As a more practical alternative, the examination of the solid-state miscibility properties at a smaller scale with a reduced number of TAG components, usually through binary or ternary mixtures, together with a deep understanding of the individual properties of the molecules involved, have been shown to deliver valuable information that may be extrapolated to real fats, such as extra virgin olive oil (EVOO) [53], cocoa butter (CB) [54,55,56], and palm oil (PO) [57,58,59,60].

**Figure 1:**
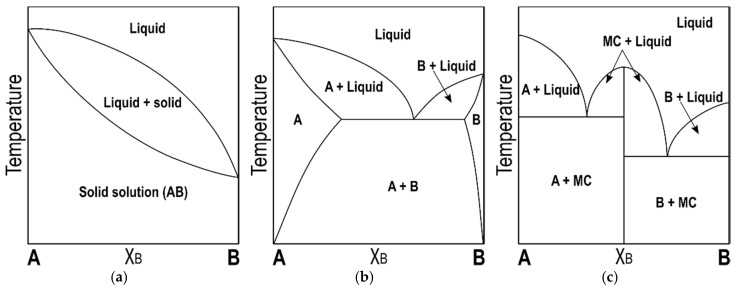
Typical phase diagrams exhibited by binary mixtures of triacylglycerols (TAGs). (**a**) Solid solution; (**b**) eutectic behavior; and, (**c**) molecular compound (MC) formation.

In order to study the mixing phase behavior of TAG mixtures, the construction of phase diagrams becomes one of the most useful and recurrent tools for obtaining a comprehensive description of the physical state as a function of temperature and percentage content of component TAGs (Χ_TAG_). In this manner, the three main types of mixing behavior that result from molecular interactions, namely eutectic behavior, complete solid solution, and molecular compound (MC) formation, can be clearly discerned through the phase boundaries at solid-solid and solid-liquid interfaces (Figure 1). 

In more detail, TAGs with equivalent thermal stability and a high degree of isopolymorphism form solid solutions at all concentration ratios, due to the ability of each TAG to randomly integrate in the crystal phase of the other without causing significant disturbance in crystal packing [61,62]. Therefore, the resulting miscible phases often present thermal and crystallographic properties halfway between those of the pure TAGs. Conversely, dissimilar TAGs lead to eutectic mixtures, in which the eutectic composition (Χ_E_) and degree of partial solid miscibility between them is largely determined by structure-derived properties, such as the type of subcell packing, chain-length structure, and melting behavior [9,60,63].

Lastly, strong interactions between TAGs that exhibit specific molecular composition and symmetry may lead to the formation of a molecular compound, whose unique structural and thermodynamic properties strongly differ from those of the individual TAGs [57,64,65,66]. Contrary to solid solutions, this kind of association presents a stoichiometric nature, which eventually results in phase diagrams with juxtaposed eutectic mixtures of the molecular compound and each component TAG bounded by the mixture at equimolecular composition.

Up to date, most of the available data concerning the solid-state miscibility of TAGs come from the study of binary mixtures under stable or near stability conditions. The very first phase diagrams are reviewed in the work of Rossell [67], and even though their accuracy has been later discussed [63,68], they still deliver valuable empirical information regarding function-molecular interactions in binary mixtures of TAGs with diverse degree of unsaturation and molecular symmetry. Many systems have been re-examined and new ones reported over time using more refined sample materials and experimental techniques, together with theoretical models for describing the phase equilibrium in TAG systems [9,61,68,69,70,71,72]. In this regard, it is worth mentioning the crucial role of synchrotron source light X-ray diffraction (SR-XRD) and Fourier transform infrared (FTIR) spectroscopy in our current understanding on the role of structural features on mixing states and the complex polymorphic behavior of TAG systems under kinetic conditions.

Bearing all of the above mentioned in mind, this paper aims to provide a brief review on the most relevant experimental data on binary and ternary mixtures of TAGs delivered over the last few decades. It should be stressed that TAGs with branched- or odd-chain fatty acids are not covered by this work, and only reports on those with even-numbered carbon atoms in their chains are discussed.

## 2. Phase Behavior in Mixtures of Fully Saturated TAGs

### 2.1. Mixtures of Monoacid Tri-Saturated TAGs

The molecular structure of monoacid saturated (Sat) TAGs is characterized by the presence of three identical constituent fatty acids esterified in the glycerol group with a “tuning fork” configuration, meaning that the two aligned fatty acids at the *sn*-1 and *sn*-3 positions of the glycerol group are oriented in the opposite direction to that at the *sn*-2 position. This leads to TAGs such as LLL, MMM, PPP, and SSS (with L, M, P, and S being lauric, myristic, palmitic, and stearic fatty acids, respectively) exhibiting the typical metastable α and β’, and stable β polymorphs packed in double chain-length structures (2L). This uncomplicated polymorphism eases the study of their solid-sate miscibility properties, largely influenced by the chain-length mismatch (∆Cn) between component TAGs and polymorph-dependent properties, such as density of packing and molecular mobility at the methyl-end region.

**Figure 2:**
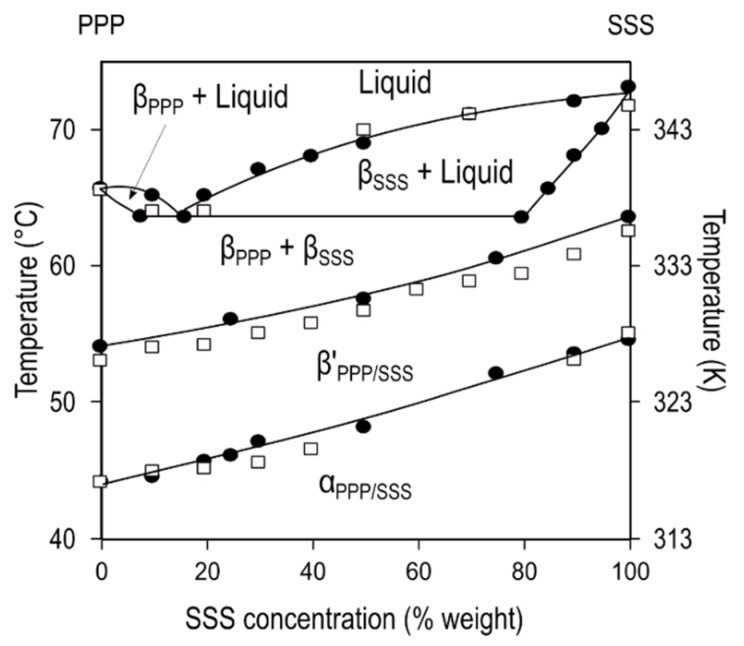
Phase diagram of PPP/SSS mixtures included in Macnaughtan et al. [75] (filled circles), with additional data from Lutton [74] (empty squares). Adapted with permission from [75], © 2006 American Oil Chemist’s Society.

The eutectic behavior of PPP/SSS (∆Cn = 2) mixtures under stable conditions was confirmed by Kerridge through the thaw-melt method [73]. Years later, laboratory-scale X-ray scattering (XRD) data provided by Lutton suggested that miscible α-2L (PPP/SSS) phases crystallized during fast cooling, which transformed via melt-mediation into β’-2L (PPP/SSS) during the subsequent heating treatment. Once the melting temperature (*T*_m_) of β’ was reached, concurrent β-2L (SSS) and β-2L (PPP) phases recrystallized [74]. Comparable results were more recently obtained by MacNaughtan et al. during the thermal processing of isothermally crystallized PPP/SSS mixtures, as depicted by the phase diagram in Figure 2 [75].

Additional research with PPP/SSS as a model system illustrated the crucial role of different factors, such as kinetic processes, thermal history, and balance of components over polymorphism and phase behavior of mixed systems. Experiments at varying cooling rates suggested that, below 20 °C·min^−1^, the homogeneous α-2L (PPP/SSS) split into PPP-rich and SSS-rich α phases in mixtures at Χ_SSS_ above 30%, with the effect being more pronounced at a decreasing rate [76]. The occurrence of these concentration gradients during crystallization was revealed in SR-XRD patterns of equimolecular PPP/SSS mixtures and seems to be ascribed to a hindered integration of PPP in SSS crystal phase due to limited molecular diffusion in the solid state [77]. This kinetically-driven segregation of mixed crystals is a common phenomenon observed when crystallization processes take place far from equilibrium conditions; and, therefore, it is of particular importance in the crystallization behavior of multicomponent systems [78,79].

In addition, experiments that were carried out under isothermal and slow cooling conditions (<1 °C·min^−1^) showed the growing tendency of the mixtures to form β’ crystals towards equal ratio of component TAGs in the mixtures, in contrast with the favored β crystals in pure TAGs. Similarly, the higher β’ stability in the mixed state became clear through the kinetically favored α-2L→β’-2L transformation and the delay in the subsequent β-2L stabilization during heating [76,77]. The high degree of disorder at the methyl-end group plane due to chain-length mismatch between TAGs is the likely cause of the former behavior. Thus, β’ crystallization would be favored by a less stringent methyl-end packing in the orthorhombic perpendicular subcell (O⊥), whereas steric hindrance in the tightly packed triclinic parallel (//T) subcell may lead to a reduction in the driving force for the β’-2L→β-2L transformation [76,80,81].

**Figure 3:**
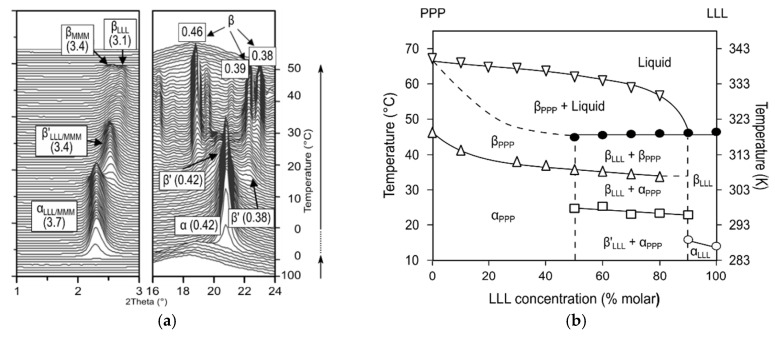
Mixing phase behavior of binary mixtures of monoacid tri-saturated TAGs. (**a**) Synchrotron source light X-ray diffraction (SR-XRD) patterns of a 70LLL/30MMM mixture during a thermal process of cooling and subsequent heating at 100 °C·min^−1^ and 5 °C·min^−1^, respectively; and, (**b**) kinetic phase diagram of LLL/PPP mixtures. Adapted with permission from [70,82], © 2020 John Wiley & Sons, © 2003 American Chemical Society.

Later, Takeuchi et al. used small- (SAXD) and wide-angle (WAXD) synchrotron X-ray scattering (SR-XRD) in order to evaluate the mixing phase behavior of the binary systems LLL/MMM, LLL/PPP, and LLL/SSS during a similar thermal process of cooling and subsequent heating at the rates of 100 °C·min^−1^ and 5 °C·min^−1^, respectively (Figure 3). In LLL/MMM, with the lowest ∆Cn (=2) of the mixtures under study, the presence of the characteristic α peak at 0.42 nm and the single diffraction peak at 3.7 nm confirmed the formation of a miscible α-2L (LLL/MMM) phase at all concentration ratios at the end of the crystallization step [82] (Figure 3a). On heating, the changes in diffraction peaks indicated the solid-state transformation of α-2L (LLL/MMM) into β’-2L (LLL/MMM), which, as observed in the PPP/SSS system, eventually decomposed in eutectic β-2L (LLL) and β-2L (MMM) forms. Therefore, it seems that the miscibility of metastable phases in monoacid TAGs may be favored by low differences in the length of acyl chains (∆Cn = 2).

Conversely, the increase of chain-length mismatch in LLL/PPP (∆Cn = 4) and LLL/SSS (∆Cn = 6) binary mixtures resulted in completely different behavior with no miscibility in any of the polymorphic forms. Moreover, the corresponding phase diagrams became significantly complex, exhibiting distinct crystallization and mixing phase behavior domains as a function of mixtures composition (Figure 3b). For both systems, the absence of diffraction peaks associated to LLL in mixtures at Χ_LLL_ below 50% suggested its dissolution in the solid fraction of the high melting TAG (PPP, SSS) during the crystallization step. Thus, α-2L crystals rich in PPP (or SSS) were obtained at the end of the cooling step, which, upon the following heating treatment, transformed into stable β-2L forms without passing through the intermediate β’-2L. In mixtures at a Χ_LLL_ above 50%, the concurrent crystallization of β’-2L (LLL) and α-2L (PPP, SSS) forms during cooling and the separately β’-2L→β-2L (LLL) and α-2L→β-2L (PPP, SSS) transformation events upon heating evidenced the great immiscibility of the systems in both metastable and stable states. In the particular case of the LLL/PPP system, a third domain was observed at a very low concentration of the high melting TAG. The total absence of PPP diffraction peaks along the whole thermal processing in the 90LLL/10PPP mixture suggested that the high melting TAG integrated in the crystalline phase of LLL, which ultimately determined the overall behavior of the mixture [82]. Moreover, the shifting of Χ_E_ from a more symmetric to a very asymmetric composition in the LLL/MMM→LLL/PPP→LLL/SSS direction highlighted the strong influence of ∆Cn (∆*T*_m_) on the eutectic behavior in the stable β forms.

The polymorph-dependent mixing behavior exhibited by the mixtures may be thermodynamically explained through the expected degree of disturbance at the methyl-end level during the formation of a mixed crystal (Table 1). Molecular packing of TAGs in the hexagonal subcell (α) is characterized by a high degree of mobility at the methyl-end region due to the disordered disposition of acyl chains in a similar manner to lamellar structures of liquid crystals [83]. Thus, the accommodation of distinct methyl-end groups may be facilitated in the miscible α-2L (PPP/SSS) and α-2L (LLL/MMM) phases due to a very low near zero excess Gibbs free energy of mixing at ∆Cn = 2 [61,68]. Although in a lesser degree, some mobility is expected to remain in the orthorhombic perpendicular subcell that would also clarify the occurrence of miscible β’ phases. However, larger methyl-end stacking gaps at increasing ∆Cn result in an increase of entropy that may lead to phase demixing in LLL/PPP and LLL/SSS systems for any of the polymorphs involved [70,84]. A different case is the triclinic parallel subcell (β), in which the dense molecular packing prevents from the crystallization of non-isomorphic TAGs in the same crystal lattice. In this connection, a recent study, based on experimental data and computer simulations, associated the β immiscibility of PPP/SSS mixtures with the steric effect induced by the protrusion of large SSS end-chains into the interface between adjacent lamellas [85]. By taking this into account, it becomes evident the strict requirements of molecular resemblance and thermal behavior of solid solution-forming TAGs, which might be alleviated to some extent in the less stable crystalline packing. Thus, it is not surprising the more prevalent mixing phase behavior of eutectic nature in TAG systems found in the scientific literature.

### 2.2. Mixtures Including Mixed-Acid Tri-Saturated TAGs

Table 2 [59,72,86,87,88,89] summarizes the polymorphic forms encountered in main mixed-acid tri-saturated TAGs. The noticeable higher complexity in polymorphism as compared to monoacid TAGs arises from the effect of chain length mismatch between adjacent fatty acids on methyl-end stacking [28,90,91,92,93,94,95]. Typically, α, multiple β’ and β forms having double chain-length structures are found, although diverse β-3L may occur, depending on the length of the constituent fatty acids and the characteristic methyl terrace [95]. In addition, polymorphic occurrence during crystallization processes has shown strong structural dependence. In this context, the distinct crystallization tendency exhibited by SSS (β), PSS (β’) and PPS (α) has been related to the degree of structural disturbance at the methyl-end planes at increasing number of missing -CH_2_ groups [96]. Regarding polymorphic stability, asymmetric TAGs tend to stabilize in its β’ form and β forms are hardly obtained [97,98,99,100]. For symmetric TAGs, β polymorphs with a “tuning fork” configuration of glycerol groups are frequently encountered, but resilient β’ may still occur in some cases for some specific molecular structures. Thus, β’ most stable forms in TAGs with a C_n_C_n + 2_C_n_ structure (n = even C number), such as CLC (with C being capric acid), LML, MPM, and PSP have been related to their “chair” type configuration, which may not undergo a further solid-state transformation into “tuning fork” type β forms [86,101,102,103]. The importance of the polymorphic occurrence briefly discussed above (for a more detailed description the readers should refer to recent book chapters [28,70,104]) is ultimately revealed on the phase behavior of mixed-acid TAGs and ensuing thermophysical properties shown by complex fats, like the β’ stable coconut oil [15,105,106].

Research carried out in binary mixtures of monoacid and mixed-acid tri-saturated TAGs has shown the predisposition of these systems to display mixing phase behavior of eutectic nature, and a major influence of the mixed-acid component on the overall polymorphic behavior during crystallization, transformation, and melting events. The main features of binary mixtures of TAGs with such configuration will be illustrated through the kinetic phase behavior that was reported for SSS/PSS, PPP/PSS, and PPP/PSP binary systems [72,107,108].

All three systems exhibited metastable α phases at the end of fast crystallization processes, but the small changes in TAGs composition and configuration led to distinct effects of kinetic and compositional factors when moderate-slow rates were applied. The former was revealed during the cooling of SSS/PSS mixtures at 0.1 °C·min^−1^: PSS crystallized in its β’-2L form at all compositions, whereas the β-crystallization tendency of pure SSS decreased, since only α-2L (SSS) crystals were observed at a Χ_PSS_ of 40% and above [72]. Altogether, the thermal behavior during the subsequent heating step was not influenced by crystallization conditions and Χ_E_ remained constant around the equimolecular composition. In addition, melting data on mixtures around a Χ_PSS_ of 40% suggested an enhanced β’ occurrence, which was presumably due to the formation of SSS-PSS bilayers after melting of α-2L (SSS).

**Figure 4:**
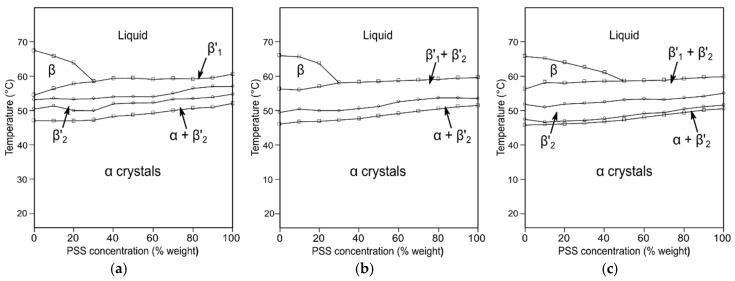
Kinetic phase diagrams of PPP/PSS system obtained under different kinetic conditions. (**a**) Cooling at 10 °C·min^−1^ and heating at 5 °C·min^−1^; (**b**) cooling at 1 °C·min^−1^ and heating at 5 °C·min^−1^; and, (**c**) cooling and heating at 1 °C·min^−1^. Adapted with permission from [107], © 2018 Wiley-VCH.

As for PPP/PSS and PPP/PSP mixtures, the effect of small structural changes on the crystallization behavior was evident during cooling at 1 °C·min^−1^. When this cooling rate was applied, PPP/PSS behaved similarly to mixtures which were crystallized at fast rates, exhibiting α-2L phases [107]. By contrast, in PPP/PSP mixtures, α polymorphs predominated at a Χ_PSP_ below 30%, whereas mixtures at increased PSP content were characterized by the concurrent presence of β’_2_ and β’_1_ crystalline phases [108]. The kinetic phase diagrams of PPP/PSS mixtures that are displayed in Figure 4 may serve as an example of the mixing behavior in binary systems of monoacid and mixed-acid fully saturated TAGs. Overall, the sequence of polymorphic transformations starting from the crystallized α phase was not influenced by the thermal history of the mixtures or the heating rates applied [107]. For all of the mixtures under the different conditions, the melt-mediated transformation from α to β’_2_ phase was followed by its solid-sate transformation to β’_1_ phase, which showed to be the most stable in the mixtures, except for those that are rich in PPP, in which the additional recrystallization of a β phase after β’_1_ melting occurred. In addition, the results suggested that the most stable form obtained in the mixtures before complete melting varied as a function of kinetics, as well as the symmetry of the eutectic composition in the phase diagram. Accordingly, during the heating process at 5 °C·min^−1^, the PPP/PSS system showed to be eutectic at a Χ_PSS_ of 30%, and β-stable mixtures were only observed below this value, independently of the cooling rate applied upon crystallization (10 and 1 °C·min^−1^). Conversely, when the heating rate was decreased to 1 °C·min^−1^, Χ_E_ shifted to the 50PPP/50PSS composition and β polymorphs were still visible at a Χ_PSS_ of 40%, which is in great agreement with the kinetic phase behavior reported for SSS/PSS mixtures [72]. In a similar manner, distinct β and β’ stable phases were found in PPP/PSP mixtures at each side of the Χ_E_, which, in this case, remained at a constant Χ_PSP_ of 30%, regardless of the different thermal processing applied [107].

In broad terms, these studies underlined how mixed-acid TAGs influence kinetics in such a way that, even at low concentrations, seem to dictate the overall polymorphic behavior during crystallization and transformation by easing the formation of β’ phases and hindering the β’→β transformation [59,72,107,108]. The apparent dominating role of mixed-acid TAGs in mixtures with monoacid TAGs is likely determined by the disturbing effect of chain-length mismatch over methyl-end group packing within interlamellar regions.

The effect of chain-length mismatch, kinetics, and mixture composition over crystallization, melting, and mixing phase behavior of fully saturated TAGs was clarified by Narine’s group through the DSC and laboratory-scale XRD study of binary systems exhibiting positional isomerism. In more detail, tri-saturated TAGs constituted by C, L, M, P, and S fatty acids were combined by pairs in symmetric/asymmetric units with an ABA/AAB configuration and examined during a thermal processing consisting of cooling the melt at varying rates (3 °C·min^−1^ and 0.1 °C·min^−1^) and subsequently heating at 5 °C·min^−1^ [104].

During the crystallization step, TAG molecules tended to rearrange in more ordered structures at slower cooling and increasing chain-length mismatch. Thus, crystallization behavior at ∆Cn = 2 in PSP/PPS (α crystals) was independent of the cooling rate [109], whereas, in MSM/MMS (∆Cn = 4), the crystallization tendency changed from α to β’ when the cooling rate was decreased [110]. Higher difference in fatty acid length resulted in a stronger influence of mixtures composition on polymorphism. LSL/LLS (∆Cn = 6) mixtures cooled at 3 °C·min^−1^ showed concurrent α and β’ crystals, with the second being particularly predominant at a Χ_LSL_ of 30% and above, which was likely due to a stabilizing effect of the symmetric TAG over LLS [88]. Similarly, diffraction data on the CSC/CCS (∆Cn = 4) system at both cooling rates confirmed distinct β’ phases at all compositions, with the concurrent presence of β-2L crystals in mixtures at a Χ_LSL_ above 60% [89].

**Figure 5:**
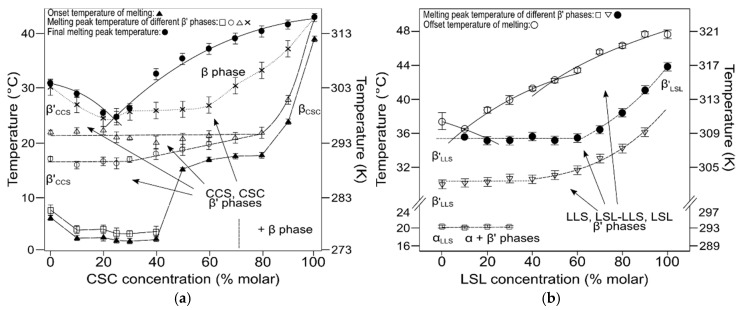
Kinetic phase diagrams of (**a**) CSC/CCS and (**b**) LSL/LLS binary systems obtained during cooling at 3 °C·min^−1^ and subsequent heating at 5 °C·min^−1^. For simplicity, the multiple β’ forms are represented by β’. Reproduced with permission from [88,89], © 2008, 2010 Elsevier.

Thermal data corresponding to the heating treatment of the mixtures and boundary analysis of the subsequent phase diagrams suggested a correlation of a larger chain-length mismatch with a higher ∆*T*_m_ between TAGs and symmetry of the eutectic composition. The liquidus curve that was obtained after the melting of β’ phases in the MSM/MMS system clarified a Χ_E_ very close to pure MMS composition. By fitting experimental results to a thermodynamic model using the Bragg-Williams approximation for non-ideality of mixing, the great immiscibility observed was ascribed to favored MSM-MSM and MMS-MMS interactions before those of MSM-MMS in both the liquid and solid-state. In CSC/CCS mixtures, the enlarged ∆Cn led to a less asymmetric eutectic diagram (Figure 5a), which was largely influenced by thermodynamic and kinetic factors. This resulted in a shifting of the eutectic point from a Χ_CCS_ of 25% at 3 °C·min^−1^ to 50% at 0.1 °C·min^−1^. Furthermore, it also became clear the more intricate polymorphic transformation behavior displayed by the mixtures when crystallization took place at a shorter time, with the effect being especially noteworthy at high concentrations of the asymmetric TAG.

As for LSL/LLS mixtures, the kinetic phase diagrams showed the presence of a singularity at a Χ_LLS_ of 50%, which consisted of a clear change of slope in the liquidus curve, delimiting two distinct eutectic regions at the LSL- and LLS-rich sides (Figure 5b). Moreover, the liquidus line that was obtained from a thermodynamic model based on the Hildebrand equation introducing a non-ideality parameter fitted well to the experimental data when the singularity was included in the calculations, as depicted by the three-segment solid-liquid transition line in the phase diagram. In agreement with previous work carried out by Perron [111,112], the same singularity was observed in the equimolecular composition of PSP/PPS mixtures. Despite the lack of confirmation by diffraction data for either PSP/PPS or LSL/LLS systems, such a complex behavior might be ascribed to the formation of a molecular compound at the 1:1 concentration ratio [57,64,113]. These results were in great contrast with the very asymmetric eutectic diagrams reported for the PSP/PSS system [107], and highlighted the influence of small changes in the fatty acid composition and distribution on the phase behavior of mixed-acid TAGs (see Table 3). The formation of fully saturated crystal compounds was also suggested by Knoester et al. in the SSP/SPS and PPS/PPP systems on the basis of clear point data, which, at equimolecular composition, were substantially above the liquidus boundaries [114]. The dense intermolecular packing ascribed to molecular compounds may be favored by shape factors in specific combinations of symmetric/asymmetric tri-saturated TAGs. However, no further efforts have been made to unveil the specific structural features.

As shown by binary mixtures of TAGs including unsaturated (Unsat) fatty acid moieties (further explained in next sections), chain–chain interactions and glycerol conformation are also expected to exert a crucial role on solid-state miscibility properties of fully saturated systems. The physical properties of mixed-acid TAGs and their mixtures (high melting point, oxidation resistance, β’ stability) make them of interest for many food technological applications, and the structure-function relationships are still, therefore, a fundamental field for further research and discussion.

## 3. Phase Behavior in Mixtures of Saturated-Unsaturated Mixed-Acid TAGs

### 3.1. Mixtures of Saturated-cis-Unsaturated Mixed-Acid TAGs

Table 4 [24,25,42,46,64,115,116,117,118,119,120] summarizes the polymorphic forms, including transient phases, such as liquid crystalline structures (LC), encountered for the main saturated-*cis*-unsaturated mixed-acid TAGs that are included in the mixtures under review. Along with the characteristic methyl-end stacking and glycerol conformation, their polymorphism (subcell packing, number of forms and subforms, chain-length structure) becomes remarkably complicated by the steric hindrance between saturated and unsaturated acid moieties, with the latter usually being oleic acid (O). Likewise, intermolecular Sat-Sat, Unsat-Unsat, and Sat-Unsat chain interactions influence the lateral packing in mixed TAG systems and, hence, their tendency to form either solid solutions, eutectic mixtures, or molecular compounds.

The formation of molecular compounds between TAGs at a 1:1 concentration ratio was first suggested by Moran for the POP/OPO system. The diffraction patterns of thermodynamically stabilized mixtures showed the increasing presence of a novel β-2L structure towards equivalent mass ratio of TAGs, in contrast with the typical β-3L stability of pure POP and OPO component TAGs [121]. Rossell suggested similar behavior for SOS/SSO mixtures on the basis of melting behavior and infrared spectroscopy data provided by Freeman [122] and Chapman [123], which was subsequently confirmed by Engstrom in a systematic study of TAG mixtures based on S, P, and O fatty acids [124]. Moreover, the formation of crystal compounds suggested in SOS/PSO, SOS/SPO, SOS/PPO, and POP/PPO systems indicated that this type of mixing behavior seemed to be restricted to some mixtures with a specific Sat-O-Sat/O-Sat-O and Sat-O-Sat/Sat-Sat-O configuration.

Years later, Sato’s group carried out a more detailed study of the molecular compound-forming systems SOS/SSO, SOS/OSO, POP/PPO, and POP/OPO, with special emphasis on the polymorphism and mixing phase behavior under dynamic thermal conditions [57,64,113,115]. In addition, the role of chain-chain interactions, glycerol conformation, and methyl-end stacking in the structural stabilization was deeply investigated by implementing SR-XRD and FT-IR techniques. [65,125,126].

**Figure 6:**
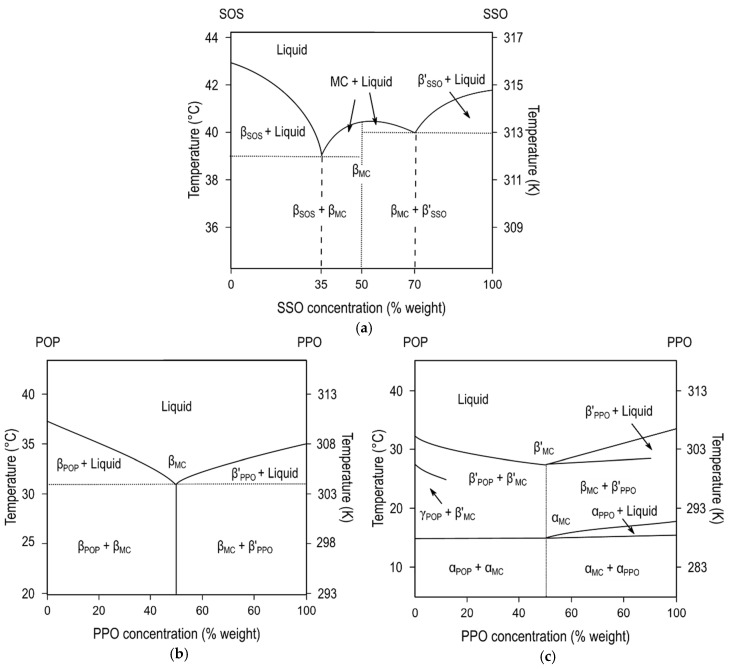
Phase diagrams of molecular compound-forming systems. (**a**) Stable diagram of SOS/SSO mixtures. Reprinted with permission from [115], © 2002 Elsevier. (**b**) Stable and (**c**) kinetic phase diagrams of POP/PPO system. Reprinted with permission from [57], © 1997 American Chemical Society.

Figure 6a depicts the phase diagram of SOS/SSO mixtures in the most stable forms, obtained after thermodynamic stabilization [115]. The formation of molecular compound crystals with a maximum *T*_m_ was detected at the 50SOS/50SSO composition, whereas the two depressions in melting point at a Χ_SOS_ of 35% and 70% showed the respective β-3L (SOS)/β-2L (MC_SOS/SSO_) and β-2L (MC_SOS/SSO_)/β’-3L (SSO) eutectic mixtures at the SOS- and SSO-rich regions of the diagram. Conversely, no increase in the *T*_m_ of β-2L (MC) was observed between the juxtaposed eutectic regions in POP/PPO, POP/OPO, and SOS/OSO binary mixtures [57,64,113] (Figure 6b).

DSC and SR-XRD experiments that were carried out on these systems under kinetic conditions confirmed the occurrence of molecular compound metastable forms, also exhibiting immiscible behavior with pure component phases. During the heating of the POP/PPO mixture at a 1:1 concentration ratio, α (MC_POP/PPO_) transformed into β’ (MC_POP/PPO_) via melt-mediation [57] (see phase diagram in Figure 6c), whereas in POP/OPO α (MC_POP/OPO_) directly transformed into β (MC_POP/OPO_) through the solid state [64]. In addition, the strong influence of molecular compounds on the crystallization and polymorphic transformation behavior of TAG mixtures became evident, especially in POP-rich regions of the diagrams. The α-2L→β’-2L transformation of POP through an intermediate γ-3L was hindered, and the β_2_-3L→β_1_-3L was retarded at low concentration levels of MC_POP/PPO_ in POP/PPO mixtures. As for the POP/OPO system, the stabilization of POP was remarkably accelerated through a promoted β’-2L (POP)→β-3L (POP) transition in the presence of MC_POP/OPO_.

**Figure 7:**
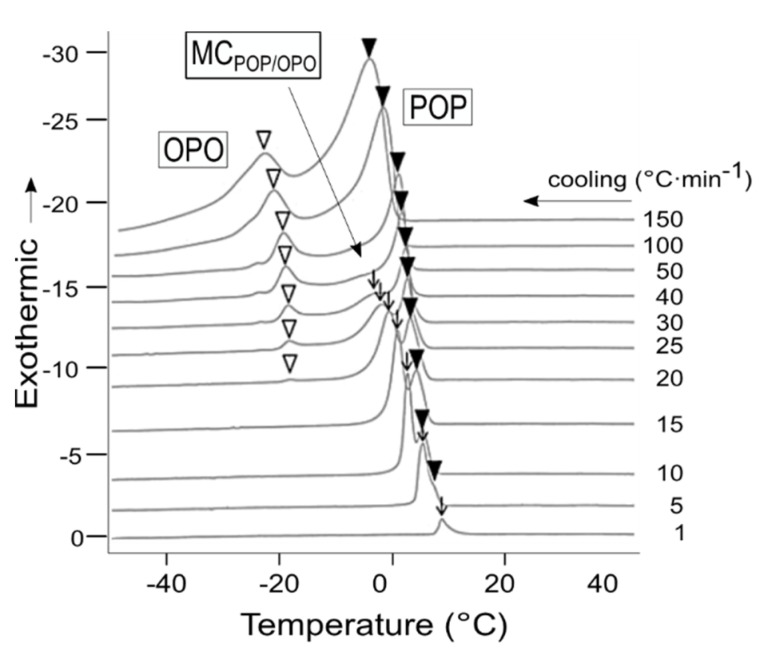
Crystallization behavior of 50POP/50OPO mixtures from low to very high cooling rates. Adapted with permission from [47], © 2018 American Oil Chemist’s Society.

The crystallization and mixing phase behavior of molecular compounds is particularly relevant in industrial processes, as they may condition the efficient separation of TAGs during oil fractionation [10] or the functionalities of lipidic materials obtained through fat blending. In this connection, molecular compounds have been suggested as alternative structuring agents to increase oleic acid and reduce saturated and *trans* fats content in food products [11]. Thus, valuable information for fat systems can be obtained from TAG mixtures when close to real manufacture conditions are employed. For example, the application of varying cooling rates (1 to 150 °C·min^−1^) has shown to influence the crystallization behavior of POP/OPO mixtures in a way that the single crystallization of molecular compound is limited to relatively slow cooling, whereas the system tends to complete immiscibility when the rate is sufficiently increased [47] (Figure 7).

In an attempt to illustrate the behavior of molecular compound-forming systems under conditions resembling those of edible fats and oils fractionation processes, the work of Minato et al. on MC_POP/OPO_ and MC_POP/PPO_ crystals grown from neat liquid [57,64] was further developed by a study on the mixing behavior of POP/OPO and POP/PPO mixtures in *n*-dodecane (at 20% and 50% solution) [127,128]. Overall, crystallization and melting temperatures were affected by the amount of solvent in the mixtures due to solubility effects. However, the specific interactions between TAGs were so strong that the solvent did not affect the formation of molecular compounds, even at levels up to 98%. Moreover, the single phases of molecular compound obtained during cooling the equimolecular mixture of POP/OPO at 2 °C·min^−1^ and 5 °C·min^−1^ indicated its favored crystallization at the expense of pure components, which was strongly influenced by solubility properties and the rates of nucleation and crystal growth. In line with this, Bayés-García et al. used synchrotron radiation microbeam X-ray diffraction (SR-μ-XRD) to clarify the competitive crystallization of POP, OPO, and MC_POP/OPO_ through the microstructure of spherulites composed by 75POP/25OPO and 25POP/75OPO mixtures, which were crystallized under selected supercooling conditions [129]. In POP-rich spherulites, the inner part was dominated by β (MC_POP/OPO_) crystals, whereas β-3L (POP) form predominated towards the outer region. This indicated that the rate of nucleation of the molecular compound was higher than that of β-3L (POP) at the experimental conditions examined. By contrast, the uniform microstructure mostly composed by β-3L (OPO) in OPO-rich spherulites suggested the similar or slightly higher nucleation rate of OPO compared to that of MC_POP/OPO_. Additional extensive studies by using SR-μ-XRD may shed more light on the crystallization kinetics and growth mechanisms of TAG mixtures and complex fat systems.

**Figure 8:**
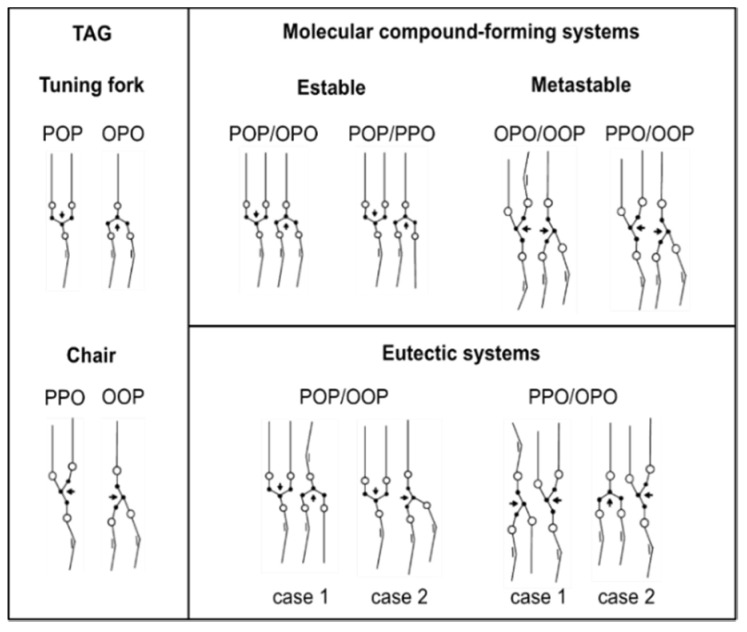
Proposed structural models for saturated-*cis*-unsaturated TAGs and their binary mixtures. Reprinted with permission from [130], © 2015 American Chemical Society.

The formation of molecular compound in saturated-unsaturated mixed-acid TAGs may be more easily understood by considering the corresponding molecular structure models for pure TAGs and analyzing the stabilizing or destabilizing molecular interactions between them, as depicted in Figure 8 [57,64,118,130]. In this regard, it is worth mentioning the close structural properties between molecular compounds ascribed by Minato et al. to the pairs SOS/SSO and POP/PPO, and SOS/OSO and POP/OPO [65], which allow foreseeing an equivalent nature of the molecular interactions that are involved in their stabilization.

It is assumed that glycerol groups in symmetric (achiral) TAGs tend to adopt a “tuning fork” configuration, whereas asymmetric (chiral) TAGs exhibit the “chair” configuration [131]. Subsequently, fatty acid chains at the *sn*-1 and *sn*-3 positions are set in an opposite direction to that of the *sn*-2 position in POP and OPO, whereas fatty acids located at the *sn*-1 and *sn*-2 positions are directed in opposite turn to that located in the *sn*-3 position in PPO and OOP. This arrangement of the fatty acid chains contributes to the molecular stability by avoiding steric hindrance between palmitoyl and oleoyl chains. Thus, the predicted structure for MC_POP/OPO_ with an opposite turn of the neighboring glycerol groups and separated saturated and unsaturated leaflets seems plausible, and steric hindrance emerges as the driving force for crystal packing in a double chain-length structure. In addition, the close contact between glycerol and methyl-end groups assumed in stable β-3L forms of POP and OPO and the subsequent excess energy for molecular packing would be prevented in the molecular compound. This is expected to contribute to the reported higher crystallization rate of MC_POP/OPO_ before pure POP [128].

The stability of MC_POP/PPO_ might be more difficult to explain, since the unbalanced content in palmitic and oleic fatty acids may inevitably lead to the presence of adjacent palmitoyl and oleoyl chains in one of the leaflets. According to FT-IR data on MC_POP/PPO_, the resulting steric effect seems to be responsible for the deviation in the olefinic conformation from the typical skew-*cis*-skew’ (s-*c*-s’) and skew-*cis*-skew (s-*c*-s) types [65]. However, the destabilizing effect of palmitic-oleic interactions could be compensated if a structure with both TAGs in a “tuning fork” configuration is assumed. The more favorable disposition of glycerol groups in parallel arrangement and with an opposite turn, together with affinity interactions in the palmitoyl leaflet, might be decisive in the stable nature of MC_POP/PPO_.

Similarly, chain–chain interactions and glycerol conformation seem to play a major role on the singular mixing phase behavior reported for the PPO/OOP and OPO/OOP systems. Monitoring the mixtures during thermodynamic stabilization over a 17-month period confirmed that both of the systems were able to form metastable molecular compounds, but displayed eutectic behavior once stability was reached. In PPO/OOP mixtures, the decomposition of the molecular compound into constituent TAGs over time was confirmed by the decrease of intensity of diffraction peaks corresponding to a molecular compound β’-2L structure at the expense of those of β’-3L (PPO) and β’-3L (OOP) [130]. As for the OPO/OOP system, a preceding β’ (MC_OPO/OOP_)→β (MC_OPO/OOP_) transformation could be identified before phase demixing took place. The occurrence of molecular compound crystals in this system may be favored by the stable oleoyl leaflet and the parallel disposition of neighboring glycerol groups assuming a “chair” configuration of OPO (Figure 8). However, a transformation to a more stable “tuning fork” glycerol configuration during thermodynamic stabilization may increase the steric hindrance, thus explaining the metastable nature of the molecular compound.

**Figure 9:**
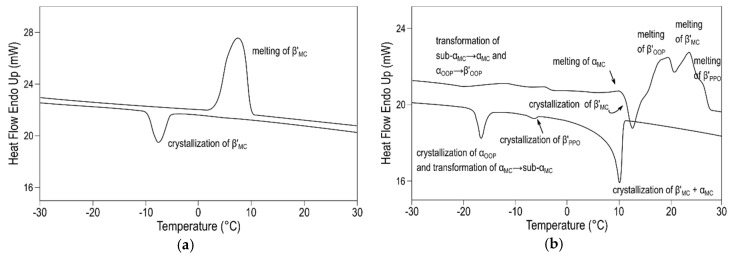
Polymorphic behavior in equimolecular mixtures of (**a**) OPO/OOP during cooling at 0.5 °C·min^−1^ and subsequent heating at 2 °C·min^−1^; and, (**b**) PPO/OOP during cooling and heating at 2 °C·min^−1^. Reprinted with permission from [130], © 2015 American Chemical Society.

Additional experiments under varying cooling and heating conditions allowed for confirming the further occurrence of a sub-α form of MC_OPO/OOP_ and sub-α and α forms of MC_PPO/OOP_. As examples, Figure 9a and b depict the DSC thermograms that were obtained for these two molecular compounds under different thermal protocols. During cooling at 0.5 and 2 °C·min^−1^, the single peak displayed in 50OPO/50OOP cooling thermograms indicated that the system readily crystallized in a β’ (MC_OPO/OOP_) form, thus leading to quite simple behavior upon melting. By contrast, the 50PPO/50OOP mixture exhibited the crystallization of α (MC_PPO/OOP_), β’ (MC_PPO/OOP_), and pure TAGs during the cooling process at 2 °C·min^−1^, which was followed by complex polymorphic transformation processes during the subsequent heating step. Such a complicated polymorphic behavior might be indicative of competitive S-OPP/R-PPO, S-OOP/R-POO, and (R-)S-OPP/(R-)S-OOP interactions during the molecular arrangement of TAGs. Therefore, the racemicity exhibited by both TAGs could be the underlying cause of the metastability of MC_PPO/OOP_, hardly explained just by means of the proposed molecular structure [130] (Figure 8).

In view of the above, the stereochemistry of asymmetric TAGs becomes an additional factor to be considered when it comes to solid-state miscibility of multicomponent TAG systems. It seems relevant to gain a clear picture about the polymorphic behavior of chiral TAGs in both their pure enantiomeric forms and in racemic mixtures. Different studies on asymmetric TAGs with diverse chain-length mismatch and degree of unsaturation, such as LLM, PPM, or POS [99,132,133], concluded that enantiomers tended to β’ stability, whereas racemates showed stable β forms. This was explained by Craven & Lencki through a model of the relative stereochemistry in unit cells, based on the available data on crystalline tendency and space group determination in asymmetric TAGs. Accordingly, unit cells of β’ polymorphs may be enantiomerically pure, whereas those of β forms may be stereochemically mixed with the formation of a racemic compound [131,134].

However, mixtures of S-OPP and R-PPO were shown to come into conflict with the above mentioned, as no stable β forms were obtained at a 1:1 concentration ratio after thermodynamic stabilization [57]. Moreover, most stable form for both the enantiomeric forms and the racemic mixture was β’-3L [117]. The concurrent polymorphic crystallization and transformation of pure enantiomers and racemic compound when cooling and heating S-OPP/R-PPO mixtures (10% concentration intervals) at 2 °C·min^−1^ stated the eutectic nature of the system. In addition, the R and S enantiomers exhibited similar thermophysical properties, and these differed from those of the racemic mixture, which showed lower crystallization and melting temperatures. During cooling, S- and R-PPO crystallized in β’_1_-3L form, and complementary experiments at varying cooling conditions confirmed that the least stable α_2_-2L form only occurred when rates above 10 °C·min^−1^ were applied. Furthermore, the SR-XRD patterns of the equimolecular composition agreed with the reported tendency of racemic mixtures to crystallize in α forms [135]. The occurrence of the two distinct α_2_-2L and α_1_-3L forms at the end of the cooling process, and their respective α_2_-2L→β’_1_-3L polymorphic transformation, for S and R enantiomers, and the sequence α_1_-3L→β’_2_-2L→β’_1_-3L, for the racemic mixture; during heating proved that, even at equal ratio of enantiomers in the mixture, not all of the molecules contributed to the formation of the racemic compound [117]. Although a few steps have already been taken to assess the effect of stereochemistry in mixed TAGs systems [116,118], there is still a considerable gap in our knowledge regarding the influence of chiral TAGs in either the enantiomeric form or racemic mixture on the structure-function properties of lipid blends.

As previously stated, in the absence of specific interactions that allow for a close molecular packing, the presence of kink sites arising from *cis* double bonds may contribute to the immiscibility of TAGs systems by causing a disruption during crystal packing. The most glaring example can be found in mixtures of tri-saturated and saturated-unsaturated mixed-acid TAGs, typically showing a very pronounced eutectic behavior with the Χ_E_ near pure composition of the lower-melting TAG.

**Figure 10:**
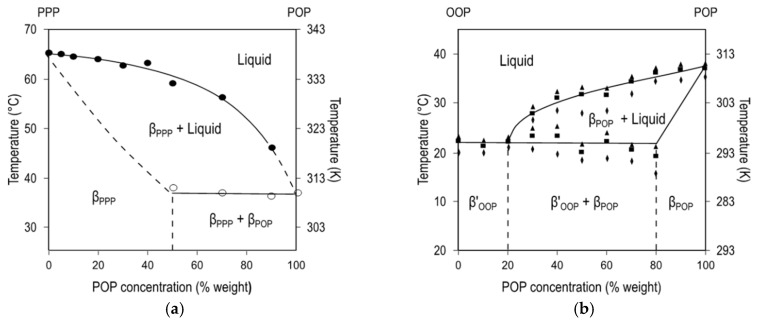
Stable phase diagrams of (**a**) PPP/POP and (**b**) POP/OOP binary systems. Adapted with permission from [60,118], © 1996, 2007 American Oil Chemist’s Society.

Figure 10a displays the phase diagram of PPP/POP binary mixtures in the most stable polymorphs. The continuous liquidus line evidenced a eutectic composition that was probably well below a Χ_PPP_ of 10%, while the solidus boundary at the PPP-rich region showed that around 40–50% of solid POP integrated in the crystalline phase of PPP. On the whole, the behavior of the system is likely due to the great ∆*T*_m_ between TAGs (∼30 °C), with the contribution of steric hindrance between palmitoyl and oleoyl chains, and the different chain-length structure of PPP (β-2L) and POP (β-3L) in the low intersolubility reported [60]. Interestingly, the kinetic phase diagram obtained after cooling at 15 °C·min^−1^ showed lower miscibility of the system in the less stable α form, which agreed with previous results obtained by Gibon et al. after melting and quenching at 25 °C·min^−1^ [59]. However, this behavior seems to be ascribed to kinetic effects, since a more recent study carried out at a lower cooling rate suggested that only α forms of PPP might be present in mixtures at a Χ_PPP_ of 60% and above [58], which is more in accordance with the favored solid-state miscibility attributed to metastable phases.

Similar results, which consisted of a eutectic behavior with a very asymmetric Χ_E_, were reported for PPP/OOP mixtures, as expected from the higher ∆*T*_m_ caused by the additional oleoyl chain in OOP [58,68]. Furthermore, the integration of OOP in the crystal lattice of PPP was noticeably lower (∼15%) than that of POP, which suggested a greater disturbance during crystallization at increasing number of olefinic groups. Understandably, equivalent behavior of immiscible nature was displayed by mixtures of monoacid saturated and unsaturated TAGs, such as PPP/OOO and SSS/OOO [67,74,136].

As for binary systems in which both TAGs present at least one unsaturated fatty acid moiety, the occurrence and complexity of the eutectic behavior is especially sensitive to small changes in the fatty acid distribution at the *sn*-positions. Contrary to the stable molecular compounds that were reported for POP/OPO and POP/PPO, and the metastable ones of PPO/OOP and OPO/OOP, the fatty acid disposition in PPO/OPO and POP/OOP systems led to immiscibility between component TAGs under stable and metastable conditions [118,130]. Moreover, while the PPO/OPO system exhibited very simple eutectic behavior with a Χ_E_ near pure OPO, POP/OOP mixtures resulted in a peritectic-type phase diagram with each component TAG being able to solubilize around 20% of the other in its solid phase (Figure 10b). Likewise, kinetic measurements on POP/OOP pointed out that crystallization and polymorphic transformation behavior of the mixtures located at the ends of the phase diagram was ruled by pure TAGs, whereas the influence of each TAG over the other became stronger towards equimolecular composition [58,118]. Very similar results were obtained in the study of SOS/*sn*-OOS, with only slight differences being ascribed to the distinct polymorphism of TAGs containing palmitic and stearic fatty acids. Therefore, the immiscibility of these systems was presumably driven by incompatible interactions involving glycerol groups and hydrocarbon chains (Figure 8) with no apparent influence of whether the chiral TAG is present in its optically active or racemic form [116].

**Figure 11:**
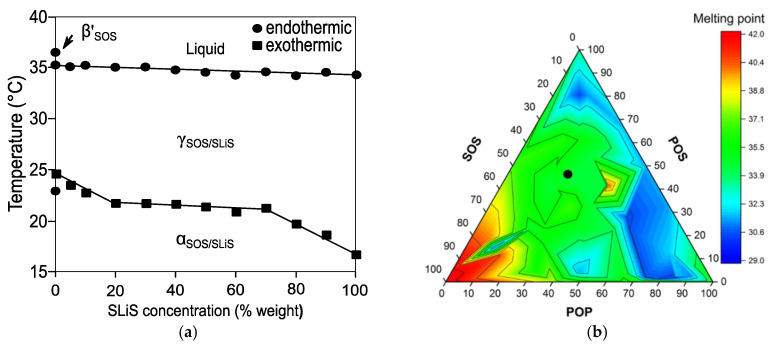
Phase diagrams of saturated-*cis*-unsaturated TAG systems exhibiting complete (SOS/SLiS) and partial (POP/POS/SOS) solid-state miscibility. (**a**) Binary phase behavior of SOS/SLiS system during heating at 2 °C·min^−1^ soon after cooling at 20 °C·min^−1^. Reprinted with permission from [137], © 2002 American Oil Chemist’s Society. (**b**) Ternary melting phase diagram of POP/POS/SOS mixtures in the β_2_ polymorph. Reprinted with permission from [55], © 2019 American Chemical Society.

The formation of complete solid solution in mixtures of saturated-unsaturated mixed-acid TAGs was revealed in the SOS/SLiS (with Li being linoleic acid) binary system. Kinetic SR-XRD experiments confirmed the formation of an α-2L miscible phase during fast cooling and its subsequent solid-state polymorphic transformation into a more stable γ-3L form when heating at 2 °C·min^−1^ [137] (Figure 11a). As pointed out for the α-2L→γ-3L transformation in pure SLiS [42], the rapid stabilization of SOS/SLiS phases in the γ-3L form was sterically favored through the separation of stearoyl and unsaturated (oleoyl and linoleoyl) leaflets by chain sorting. The absence of further transformation events at the specified conditions indicated that the typical γ-3L→β’-3L transformation of SOS [25] was prevented in the mixtures, probably due to strong chain–chain interactions hindering the separation of mixed components. However, additional experiments of cooling and rapid heating showed to affect miscibility properties of the system during the α-2L (SOS/SLS) melt-mediated transformation to more stable forms. The former became clear in mixtures at a Χ_SLiS_ below 30%, in which the concurrent recrystallization of γ-3L (SOS/SLiS) with either β’-2L or β-3L forms of SOS were indicative of phase separation. Although the specific structure-interaction relationships were left open for future research, the miscibility of the system was primarily attributed to olefinic interactions. More concretely, the disordered molecular configuration of di-unsaturated linoleoyl chains seemed to play a crucial role on the strength of molecular packing and, thus, in the phase demixing observed at low concentration levels of SLiS [137].

The implications of solid-sate miscibility of TAGs in real systems might be cleared up taking a relatively simple fat, such as cocoa butter, as a case example. With POS, SOS, and POP accounting for more than 80% of its total TAG content, thermophysical properties are dictated by their mixing phase behavior at a specific composition around 22POP/46POS/32SOS [138]. Thus, research carried out on binary and ternary mixtures of the former TAGs turned out to be useful not only for cocoa butter characterization, but also as a guide for the development of suitable lipid blends with potential use as cocoa butter alternatives.

For the POS/SOS binary system, Rousset et al. reported complete miscibility between β-3L (POS) and β_2_-3L (SOS) after several weeks of thermal incubation at room temperature [139]. By contrast, the pseudo phase diagram constructed from iso-solid lines and melting point data reported by Smith et al. suggested a possible eutectic at a Χ_POS_ around 80–90% [140]. Likewise, POP/POS and POP/SOS systems exhibited a binary eutectic diagram with respective solid-solid-liquid equilibrium points situated at a Χ_POP_ of 50% and 35%, which were very close to those that were predicted by applying the Hildebrand model. Nevertheless, more complex behavior was observed by Sasaki et al. for POP/SOS mixtures, consisting of a solid solution at a Χ_POP_ of 50% and above, eutectic at a Χ_POP_ between 45% and 20%, and a crystalline phase rich in SOS below the latter concentration [54] (see Table 5). The successfulness of the stabilization processes and the distinct experimental approaches are the likely cause of the main discrepancies that were observed among the mentioned studies.

As expected from the varying mixing phase behavior displayed by the binary systems, the study carried out on POP/POS/SOS ternary mixtures under stable conditions evidenced that the formation of either eutectic or miscible phases was strongly influenced by the balance of TAGs in the mixture. Thus, eutectic behavior predominated at Χ_POP_ and Χ_SOS_ above 15% and 35%, respectively, whereas, in mixtures at high POS concentration (Χ_POS_ ≥ 50%) and a Χ_SOS_ below 35%, miscible phases occurred [54]. Higher miscibility was revealed in POP/POS/SOS ternary mixtures in the β_2_ polymorph obtained by both solvent and melt crystallization [55] (green area in Figure 11b). The depression in melting points depicted by dark blue proved that the eutectic behavior of ternary mixtures was mainly governed by the POP/POS binary system, with the effect being particularly noticeable in the POP-rich region of the diagram. This agreed with a previous statement by Koyano et al., who pointed out the detrimental effect that POP concentration levels above those typically found in cocoa butter (denoted by a black circle in the miscible area) may have on the physical properties of lipid blends intended for being used as cocoa butter alternatives [141]. Unexpectedly, a reduced area of increased *T*_m_ was observed around the 40POP/40POS/20SOS composition, far from the SOS-rich high melting region. This exemplifies the importance of an in-depth understanding of multicomponent TAG systems in the design of versatile lipidic materials that allow for covering a wider range of applications. In this connection, a recent report by Watanabe et al. clarified the complex mixing phase behavior of SOS/SSO/OSO ternary mixtures. After 10 days of thermodynamic stabilization at 28 °C, mixtures with a 50SOS/50(SSO/OSO) composition showed to be exclusively formed by molecular compound and, more specifically, by a miscible β-2L phase of MC_SOS/SSO_ and MC_SOS/OSO_. By contrast, eutectic mixtures of MC_SOS/SSO/OSO_ and either β’-3L (SSO) or β-3L (OSO) crystals were formed when the mentioned concentration ratio was not present in the mixtures [66]. According to the SR-XRD data gathered during the cooling and subsequent heating of the mixtures at 2 °C·min^−1^, MC_SOS/SSO/OSO_ was not readily obtained from the melt, and α (MC_SOS/SSO_) and β (MC_SOS/OSO_) crystallized instead. Eventually, the stability of the SOS/SSO/OSO system in a single mixed phase was achieved once the α (MC_SOS/SSO_)→β (MC_SOS/SSO_) polymorphic transformation took place. These findings at a molecular level were further explored during the study of fat blends and dark chocolate preparations with a lipidic base composed by cocoa butter mixed with Sat-Sat-O and O-Sat-O fat fractions rich in SSO and OSO, respectively [142]. In fat blends with a constant Sat-Unsat-Sat TAG content around 50%, higher amounts of Sat-Sat-O favored the occurrence of metastable β’ (MC), whereas direct formation of stable β (MC) took place in blends rich in O-Sat-O without need of further tempering. As to dark chocolate preparations, pure cocoa butter-based chocolate-like hardness shown by samples composed by 50CB/20-30Sat-Sat-O/20-30O-Sat-O mixtures, together with the absence of fat bloom formation after one-year storage at 15–30 °C, pointed out the suitability of these specific compositions as cocoa butter equivalents.

Despite the mentioned studies, there is a great lack of recent experimental reports dealing with the solid-state miscibility properties of ternary mixtures of TAGs. A few preceding ternary phase diagrams have been reported [61,67], but the reliability of the data has been called into question and further investigation, paying special attention to the purity of the samples, stabilization procedures, and measurement techniques employed, might provide a more clear picture of the miscibility properties in multicomponent TAG systems. Therefore, basic and applied research on the mixing phase behavior of lipid mixtures with more than two components, and the interconnection with physical properties of complex fat systems, remain as an open field worthy of further exploration.

### 3.2. Mixtures of Saturated-trans-Unsaturated Mixed-Acid TAGs

Almost all the trans-fats present in food goods arise from industrial processes through the partial hydrogenation of vegetable oils, meant to confer them higher shelf life, oxidative resistance, and desired organoleptic properties, such as plasticity, pleasant mouthfeel, and high melting point. Although widely spread in the past, its correlation with a higher incidence of cardiovascular disease over the last decades has led to the pursuit of triglyceride (interesterification, use of tropical oils) and non-triglyceride (oleogelation, structured emulsions) alternatives that provide solid-like properties to edible oils in low- or zero-*trans* food products [143].

As to some examples, the structural differences between stearic (C18:0), oleic (9-*cis*-C18:1) and elaidic (9-*trans*-C18:1, E) acids primarily rely on the presence and configuration of the double bond. The kink in the acyl chain of oleic fatty acid due to the *cis* double bond becomes much less pronounced in the *trans*-isomer, according to the straighter saturated-like elaidic acyl chain. Thus, a less sterically hindered and more dense crystalline packing results in the characteristic low solubility, high density, and thermal stability of *trans*-fats [144,145]. When considering that they may account for up to 15% in some end products, understanding the structure-interaction-function properties of *trans*-TAG systems through polymorphic and mixing phase behavior studies might be useful for alternative fat structuring.

Table 6 [86,146,147,148] shows the polymorphic forms encountered for elaidic acid-based TAGs, which become slightly different to those of their saturated and *cis*-unsaturated counterparts. As an example, the *trans*-monoacid EEE exhibited crystalline α-2L and β-2L, but, contrary to SSS and OOO, no intermediate β’ forms were observed during melt crystallization or polymorphic transformation [24,149,150]. The substitution of S by E in the tri-saturated PSP and PPS did not significantly influence the molecular packing and crystal structure in the resulting TAGs PEP and PPE, which showed the respective β’_1_-2L and β-2L stable polymorphs [86,148]. As to stearo-elaidic TAGs, their greater disposition to β stability was revealed in SES, ESS, and SEE [146]. For SES and PEP, changes at the methyl-end plane as a function of the angle formed between aliphatic chains and the step plane were suggested by Van Mechelen et al. as the cause of their different polymorphic stability [147].

In stearo-elaidic binary systems, the close structural similarity between S and E seems to be determinant in the resulting mixing phase behavior. Grootscholten reported on the formation of solid solutions in SSS/SES, SSS/SSE and SSS/SEE mixtures [61,68], in contrast with the eutectic SSS/SOS, SSS/SSO and SSS/OOO systems (see Table 7 [68,148]). Moreover, mixing phase behavior near ideal miscibility was also reported for the stable SES/SSE binary system, whereas its *cis*-unsaturated equivalents SOS and SSO showed the formation of a molecular compound ascribed to strong specific interactions.

Very recently, Zhang et al. reported on the phase behavior of PEP/EPE mixtures under stable and metastable conditions, and the results were highly similar to those of the *cis*-unsaturated POP/OPO system. Thermodynamically stabilized mixtures showed the formation of a β-2L molecular compound at a 1:1 ratio, and additional SR-XRD experiments of cooling and subsequent heating at 5 °C·min^−1^ and 2 °C·min^−1^, respectively, showed the α (MC_PEP/EPE_)→β’ (MC_PEP/EPE_)→β (MC_PEP/EPE_) sequence of solid-state transformations. In the same manner, chain-chain interactions, glycerol group conformation, and methyl-end stacking were also suggested as the main stabilizers of the molecular structure [148], equivalent to that of its *cis*-unsaturated counterpart: a bilayer structure with separate saturated and unsaturated leaflets, and “tuning fork” configuration of glycerol groups arranged in parallel along the chain axis in opposite directions. Furthermore, the authors pointed out that the formation of MC_PEP/EPE_ might be energetically favored by reducing the void at PEP methyl-end plane when laterally packed with EPE. Based on these findings, the formation of molecular compounds in the structurally close PSP/SPS system was also suggested. However, this would be in contrast with a previous work based on melting point determination, which ascribed immiscible properties to mixtures of PSP and SPS [114]. Additional studies including precise structural analysis may shed some light into this matter.

## 4. Concluding Remarks

The diversity of lipid molecules, in terms of fatty acid composition and distribution, degree of unsaturation, and *cis* or *trans* configuration of double bonds, is revealed in the complex polymorphism and mixing phase behavior displayed by TAG systems. The occurrence of solid solutions, eutectic phases, or molecular compounds is the result of cooperative intermolecular interactions between acyl chains, glycerol, and methyl-end groups, whose influence over the system might differ as a function of molecular geometry. Thus, miscibility properties of monoacid and mixed-acid fully saturated TAGs seem to be largely determined by the methyl-end plane configuration. However, there are still gaps in our knowledge about the specific interactions between molecular groups that would explain some of the reported behavior, such as the formation of saturated molecular compounds. As for saturated-*cis*-unsaturated mixed-acid TAGs, significant efforts have been made to obtain structural evidence on the exhibited mixing phase behavior. Steric hindrance between saturated-unsaturated acyl chains and the disposition of neighboring glycerol groups arise as the main contributors to whether immiscible phases or molecular compounds are formed in binary mixtures of TAGs. Moreover, the great sensitivity of miscibility properties to the addition of a third component has also been highlighted in a few reports. Taking all of this into account, further reexamination of some binary systems applying powerful structure determination techniques such as SR-XRD and FTIR spectroscopy, would partly clarify the influence of molecular structure on mixing properties of TAGs systems. Furthermore, the role of external factors, such as the application of specific thermal treatments on mixing behavior should be carefully examined in order to obtain valuable information for diversified industrial applications. In this connection, giving a step forward from the more prevalent studies on binary systems to ternary or more complex TAGs mixtures might be relevant in the design of novel lipidic materials with a wider range of technological functionalities. Molecular compound crystals of saturated-*cis*-unsaturated mixed-acid TAGs might play a useful role in the development of healthier fat structuring strategies. Thus, molecular insights into their crystallization and solid-state miscibility properties in the presence of third components would result of great interest in industrial applications relating to fat blending and oil separation processes.

## Figures and Tables

**Table 1:** Phase behavior in binary mixtures of monoacid tri-saturated TAGs.

TAG System	Phase Behavior	Additional Remarks	Refs.
PPP/SSS	α and β’ miscible, β eutectic	Miscibility favored in metastable forms due to a less stringent packing of methyl-end groups	[74,75]
LLL/MMM			[82]
LLL/PPP	Eutectic	Increase of entropy at the methyl-end plane and asymmetry of Χ_E_ at higher ΔC_n_ between TAGs	[82]
LLL/SSS			[82]

**Table 2:** Polymorphic forms of main mixed-acid tri-saturated TAGs.

TAG	Polymorphic Forms	Refs.
PSS	α-2L, β’_2_-2L, β’_1_-2L, β’_0_-2L, β-2L *	[72,86,87]
PPS	α-2L, β’_2_-2L, β’_1_-2L, β-2L	[72,86]
PSP	α-2L, β’_2_-2L, β’_1_-2L	[59,86]
LSL	α-2L, β’-2L **, β-2L	[88]
LLS	α-2L, β’-2L **, β-2L	[88]
CSC	α-2L, β’_1_-3L, β’_2_-2L, β-L ***	[89]

* obtained by solvent crystallization; ** several sub-forms; *** undefined chain-length structure.

**Table 3:** Phase behavior in binary mixtures of mixed-acid tri-saturated TAGs.

TAG System	Phase Behavior	Additional Remarks	Refs.
SSS/PSS	Eutectic	Strong influence of low concentration levels of asymmetric TAGs on crystallization, polymorphic transformation and β’ stability of the mixtures	[72]
PPP/PSS			[107]
PPP/PSP	α miscible, β’ and β eutectic		[59,108]
PSP/PSS		Crystallization of more stable forms favored by the presence of symmetric TAGs. Increased symmetry of Χ_E_ at higher ΔC_n_	[107]
CSC/CCS	Eutectic		[89]
MSM/MMS			[110]
PSP/PPS	MC	Singularity in liquidus line in the form of a change of slope in mixtures at 1:1 concentration ratio	[109]
LSL/LLS			[88]

**Table 4:** Polymorphic forms in main saturated-*cis*-unsaturated mixed-acid TAGs.

TAG	Polymorphic Forms	Refs.
SLiS	Sub-α_2_-2L, sub-α_1_-2L, α_2_ *, α_1_-2L, γ-3L	[42,119]
SSO	α-3L, β’-3L	[115]
SOS	α-2L, LC1, LC2, γ-3L, β’-3L, β_2_-3L, β_1_-3L	[25,120]
OSO	α-2L, β-3L	[24]
OOS	Sub-α-2L, α-2L, LC, β’_2_-2L, β’_1_-3L	[46,116]
PPO (*sn*-PPO)	α-3L, β’_2_-2L, β’_1_-3L (α-2L, β’_1_-3L)	[117]
POP	α-2L, γ-3L, δ-3L, β’_2_-2L, β’_1_-2L, β_2_-3L, β_1_-3L	[25]
OPO	Sub-α-2L, α-2L, β’-2L, β_2_-3L, β_1_-3L	[64]
OOP	Sub-α-2L, α-2L, β’_2_-3L, β’_1_-3L	[46,118]

* transient liquid crystalline structure.

**Table 5:** Phase behavior in binary mixtures of saturated-*cis*-unsaturated mixed-acid TAGs.

TAG System	Phase Behavior	Additional Remarks	Refs.
POP/OPO	MC (α and β)	Molecular compound crystals with a double chain-length structure stabilized through glycerol groups conformation and aliphatic chain interactions	[64,121]
SOS/OSO			[113]
POP/PPO	MC (α, β’ and β)		[57]
SOS/SSO			[115,124]
SOS/SSO/OSO	Partially miscible	Mixed β phase of MC_SOS/SSO_ and MC_SOS/OSO_ crystals at a specific 50SOS/50(SSO/OSO) composition	[66]
OPO/OOP	Metastable MC	Stabilizing chain-chain interactions and/or glycerol conformation not strong enough to overcome the tendency of systems to stabilize in eutectic mixtures	[130]
PPO/OOP			[130]
S-OPP/R-PPO	Racemic MC	Eutectic mixture of racemic compound and enantiomers	[117]
POP/POS	Eutectic	Immiscible behavior ascribed to the great steric hindrance arising from the unbalanced content of saturated/unsaturated fatty acids and the different glycerol configurations	[140]
PPP/POP			[60]
PPP/OOP			[58,68]
POP/OOP			[118]
SOS/*sn*-OOS			[116]
POP/SOS	Highly miscible	Solid solution at Χ_SOS_ ≤50%, eutectic at increasing SOS	[54]
SOS/SLiS	Solid solution	Miscibility favored by the great degree of structural isomorphism between components in the mixtures	[137]
POS/SOS			[139]
POP/POS/SOS	Highly miscible	Eutectic behavior largely influenced by POP/POS	[54,55,140]

**Table 6:** Polymorphic forms in elaidic acid-based saturated-*trans*-unsaturated mixed-acid TAGs.

TAG	Polymorphic Forms	Refs.
SSE	α-2L, β’_2_-2L, β’_1_-2L, β-2L	[146,147]
SES	α-2L, β’_2_-2L, β-2L	[146,147]
EES	α-2L, β-2L	[146]
PPE	α-2L, β’_2_-2L, β’_1_-2L, β-2L	[86,146]
PEP	α-2L, β’_2_-2L, β’_1_-2L	[146]
EPE	α-2L, β’-2L, β-2L	[148]
EEP	α-2L, β’_2_-2L, β’_1_-2L	[146]

**Table 7:** Phase behavior in binary mixtures of saturated-*trans*-unsaturated mixed-acid TAGs.

TAG System	Phase Behavior	Additional Remarks	Refs.
SSS/SES	Solid solution	Integration of TAGs in the same crystal lattice favored by molecular similarity between S and E fatty acids.	[68]
SSS/SSE			[68]
SSS/SEE			[68]
SES/SSE			[68]
PEP/EPE	MC	Metastable α and β’, and stable β molecular compounds favored by aliphatic chain packing, glycerol conformation and methyl-end stacking	[148]
